# Src family kinases inhibit differentiation of intestinal epithelial cells through the Hippo effector YAP1

**DOI:** 10.1242/bio.058904

**Published:** 2021-11-15

**Authors:** Sepideh Fallah, Jean-François Beaulieu

**Affiliations:** Laboratory of Intestinal Physiopathology, Department of Immunology and Cell Biology, Faculty of Medicine and Health Sciences, Université de Sherbrooke and Centre de recherche du Centre hospitalier Universitaire de Sherbrooke, Sherbrooke, QC J1H 5N4, Canada

**Keywords:** Intestinal cell, Differentiation, Src family kinases, YAP1, TAZ, CDX2, HNF1, HNF4

## Abstract

Intestinal cell lineage differentiation is a tightly regulated mechanism that involves several intracellular signaling pathways affecting the expression of a variety of transcription factors, which ultimately regulate cell specific gene expression. Absorptive and goblet cells are the two main epithelial cell types of the intestine. Previous studies from our group using an shRNA knockdown approach have shown that YAP1, one of the main Hippo pathway effectors, inhibits the differentiation of these two cell types. In the present study, we show that YAP1 activity is regulated by Src family kinases (SFKs) in these cells. Inhibition of SFKs led to a sharp reduction in YAP1 expression at the protein level, an increase in CDX2 and the P1 forms of HNF4α and of absorptive and goblet cell differentiation specific markers. Interestingly, in Caco-2/15 cells which express both YAP1 and its paralog TAZ, TAZ was not reduced by the inhibition of SFKs and its specific knockdown rather impaired absorptive cell differentiation indicating that YAP1 and TAZ are not always interchangeable for regulating cell functions.

This article has an associated First Person interview with the first author of the paper.

## Abstract (precis)

**Summary:** Inhibition of Src family kinases leads to a sharp reduction in YAP1 expression and an increase in CDX2 and HNF4α, two regulators of intestinal cell differentiation, while its paralog TAZ appears not to be directly involved.

## INTRODUCTION

The intestinal epithelium that covers the inner layer of the mammalian intestinal lumen is characterized by its rapid renewal properties. It is composed of different cell types including crypt base columnar (CBC) stem cells, a subpopulation of quiescent stem cells, proliferating cells and post mitotic differentiated cells, all located in the crypts, which give rise to two distinct cell lineages, absorptive and secretory (Roostaee et al., 2016). Absorptive cells form about 80% of the intestinal epithelial cell population lining the villus while secretory lineages include Paneth, goblet and enteroendocrine cells (Fallah et al., 2020). Our knowledge about the mechanisms that regulate intestinal epithelial cell differentiation has improved in several aspects, although far from being completely understood.

We have shown recently that the Hippo pathway effector Yes associated protein 1 (YAP1) negatively regulates the differentiation of both absorptive and goblet cells in intestinal cell models. Knockdown of YAP1 was accompanied by an increase in the expression of caudal type homeobox 2 (CDX2) transcription factor (Fallah and Beaulieu, 2020), which is one of the master transcription factors for intestinal epithelial cell differentiation. For instance, the ectopic expression of CDX2 in undifferentiated normal rat and human crypt cells resulted in impaired proliferation and the generation of absorptive and goblet-like cells (Suh and Traber, 1996; Escaffit et al., 2006).

The Hippo pathway which restricts aberrant tissue growth, as summarized in seminal reviews (Meng et al., 2016; Boopathy and Hong, 2019; Pocaterra et al., 2020), is composed of three parts including upstream signals, a kinase core and downstream target genes. The Hippo pathway kinase core is composed of various components including mammalian STE20 kinase 1/2 (MST1/2), which with the help of the scaffolding protein WW domain-containing adaptor 45 (WW45), phosphorylates and activates the large tumor suppressor kinase 1/2 (LATS1/2). In turn, LATS1/2 kinases with the regulatory protein MOB1 phosphorylate YAP1 and its paralog transcriptional co-activator with PDZ-binding motif (TAZ) or WW domain containing transcription regulator 1 (WWTR1) in serine 127/397 and serine 89/311, respectively. Recognition and binding of phosphorylated YAP1/TAZ in S127/89 by 14-3-3 protein leads to its cytoplasmic sequestration. As well, phosphorylated YAP1/TAZ in S397/311 is further phosphorylated by casein kinase 1 delta/epsilon, which contributes to their ubiquitination and proteasomal degradation. In the absence of Hippo pathway activity, YAP1/TAZ enter the nucleus, bind to the TEA domain family member (TEADs) transcription factor and activate the transcription of genes related to cell growth. Hippo pathway activity is regulated by various upstream signals including cell–cell contact, cell polarity, mechanical signals such as stiffness and ECM composition, hormonal signals through the G-protein-coupled receptors (GPCRs) and growth factors (Meng et al., 2016; Gkretsi and Stylianopoulos, 2018; Lachowski et al., 2018; Cobbaut et al., 2020).

Interestingly, a close crosstalk between the Hippo pathway and Src family kinases (SFKs) has been reported in various cell types. SFKs are membrane-associated non-receptor protein tyrosine kinases that contain nine members including Src, Fyn, Yes and Lyn in vertebrates. In mammals, Src, Fyn and Yes are expressed in most tissues, however other members are expressed in certain cell types, mostly hematopoietic cells (Thomas and Brugge, 1997). Since Src kinase activity is repressed by phosphorylation in tyrosine 527 (Y527), dephosphorylation of Y527 by a tyrosine phosphatase leads to the intramolecular autophosphorylation of tyrosine 416 (Y416), which stimulates the kinase activity of Src (Roskoski, 2004). Given that aberrant activation of SFKs is associated with tumor development and metastasis in various cancers (Irby and Yeatman, 2000), several FDA approved drugs have been developed to target SFK proteins including dasatinib, imatinib, SFK-1 and pyrazolopyrimidine compounds (PP2) (Elias and Ditzel, 2015). In human mammary epithelial cells, which express various SFKs, Src was reported to be the predominant regulator of the Hippo signaling pathway and responsible for YAP1 nuclear localization (Kim and Gumbiner, 2015). Another study showed that the activity of YAP1/TAZ and its target genes is promoted by the activation of Src in breast cancer and melanoma cells (Lamar et al., 2019). Activation of Src through integrin-mediated adhesion resulted in LATS1/2 repression and YAP1/TAZ activation (Lamar et al., 2019). Furthermore, Src phosphorylates LATS1 in tyrosine residues, which is accompanied by the suppression of LATS1 via inhibition of its active phosphorylated sites including serine 909 and threonine 1079. Thus, inhibition of Src activity using dasatinib treatment rescues LATS1 activity (Si et al., 2017). Partial knockdown of YAP1/TAZ in mice injected with active Src (Src^Y527F^) expressing A375 cells, resulted in a reduction of Src mediated tumor growth and metastasis and extended mouse survival (Lamar et al., 2019). Nuclear localization of YAP1 was prevented in MCF-10A cells using PP2 where YAP1 phosphorylation on serine 127 (S127), was increased by PP2 treatment and the effect of PP2 was abolished by LATS1/2 knockdown indicating that the inhibitory effect of Src is on LATS1/2 kinase (Kim and Gumbiner, 2015). On the other hand, it has been shown that YAP1 nuclear retention is stimulated through YAP1 phosphorylation in the tyrosine 357 (Y357) residue by SFK in human and mouse cholangiocarcinoma cells. Treatment of these cells with dasatinib was accompanied by decreased phosphorylated YAP1 Y357 and translocation of YAP1 from the nucleus to the cytoplasm (Sugihara et al., 2018).

An association of SFKs with intestinal cell proliferation, migration and anoikis has been reported previously (Bouchard et al., 2007, 2008). Involvement of SFKs in intestinal epithelial cell differentiation has also been shown in Caco-2/15 cells, in which inhibition of SFKs with PP2 treatment led to an increase in the expression of absorptive cell markers such as sucrase-isomaltase (Seltana et al., 2013) while treatment of HT29 cells, another colorectal cancer cell line, with PP2 is accompanied by an increased level of E-cadherin and α/β/δ-catenin protein expression and strong cell–cell contact (Nam et al., 2002).

In the present study, we used the two well characterized intestinal cell lines Caco-2/15 and HT29 to study the regulatory effect of SFK on the Hippo effectors YAP1 and TAZ and intestinal epithelial cell differentiation using PP2 and dasatinib. The results show that SFK inhibition leads to a sharp repression of YAP1 expression and elevation in differentiated intestinal absorptive and goblet cell markers. Surprisingly, however, TAZ expression was not repressed in cells treated with SFK inhibitors. Furthermore, in contrast to the stimulation of cell differentiation in response to YAP1 abolition, knockdown of TAZ by a shRNA approach appeared to reduce absorptive cell marker expression. This finding indicates that YAP1 and TAZ are not interchangeable in the regulation of intestinal epithelial cell differentiation.

## RESULTS

### SFK inhibition induces differentiation toward both intestinal absorptive and goblet cell lineages

**Fig. 1.**
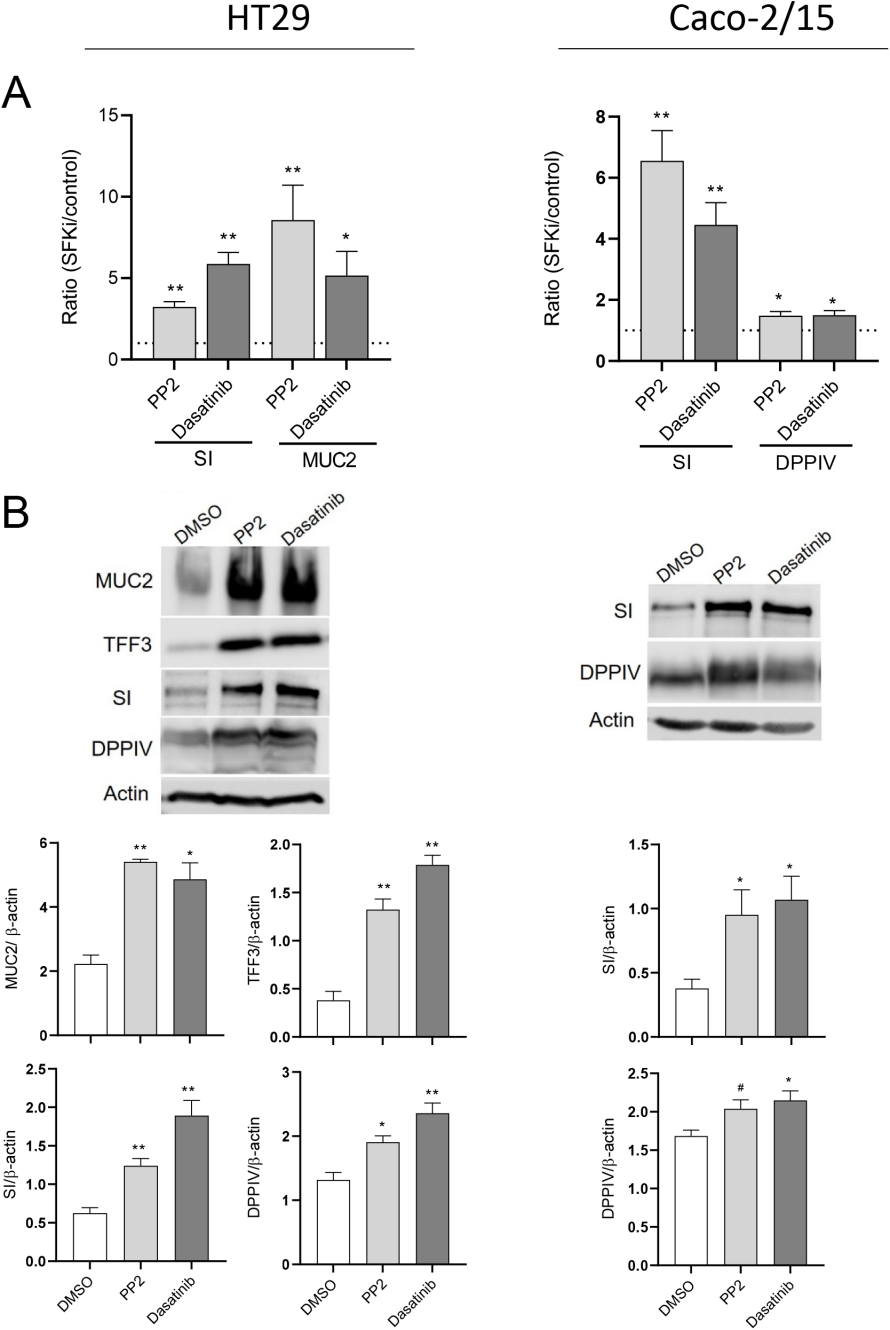
**The effect of SFK inhibition on the differentiation of absorptive and secretory cells in HT29 (left panels) and Caco-2/15 cells (right panels).** (A) Transcript expression of the absorptive cell marker SI and goblet cell marker MUC2 in HT29 and absorptive cell markers SI and DPPIV in Caco-2/15 cells incubated with SFK inhibitors (PP2 and dasatinib) relative to control (DMSO). (B) Representative western blot analysis and data compilation showing higher expression of MUC2, TFF3, SI and DPPIV in PP2 and dasatinib treated HT29 and Caco-2/15 cells relative to DMSO treated cells. β-actin was used as a loading control. Results are expressed as mean±s.e.m.; *n*=3; *P<0.05, **P<0.005, #*P*=0.063.

In order to study the effect of SFKs on intestinal epithelial cell differentiation, Caco-2/15 and HT29 cells were incubated with the SFK inhibitors (SFKis) PP2 at 20 µM and dasatinib at 10 µM with DMSO used as control. These effective concentrations of SFKi on Src activity were established for previous works for both PP2 (Nam et al., 2002; Bouchard et al., 2007; Seltana et al., 2013), and dasatinib (Kim and Gumbiner, 2015; Sugihara et al., 2018; Honeywell et al., 2020). Reduction in Src activity and increase in sucrase-isomaltase expression have been observed in post confluent Caco-2/15 cells (Seltana et al., 2013). Furthermore, it has been shown that differentiation of HT29 cells is accompanied by decreased kinase activity of Src (Park et al., 1993; Sovova et al., 1997). Treatment of the cells was started at −2 days of confluence (80% confluence) and continued for 5 days and the cells were analyzed for the expression of sucrase-isomaltase (SI) and dipeptidyl peptidase IV (DPPIV), two well characterized terminal differentiation markers for absorptive cells (Beaulieu et al., 1989; Beaulieu and Quaroni, 1991; Darmoul et al., 1992), and mucin 2 (MUC2) and trefoil factor 3 (TFF3) as specific markers for goblet cells (Kim and Ho, 2010). In HT29, expression of both absorptive and goblet cell markers was increased in cells treated with the SFKis compared with the control at both transcript and protein levels (Fig. 1, left panels). Caco-2/15 cells can only differentiate toward the absorptive lineage and both SI and DPPIV were found to be induced by SFKis (Fig. 1, right panels).

### SFK inhibition leads to YAP1 degradation but exerts no specific effect on TAZ overall expression in intestinal epithelial cells

**Fig. 2.**
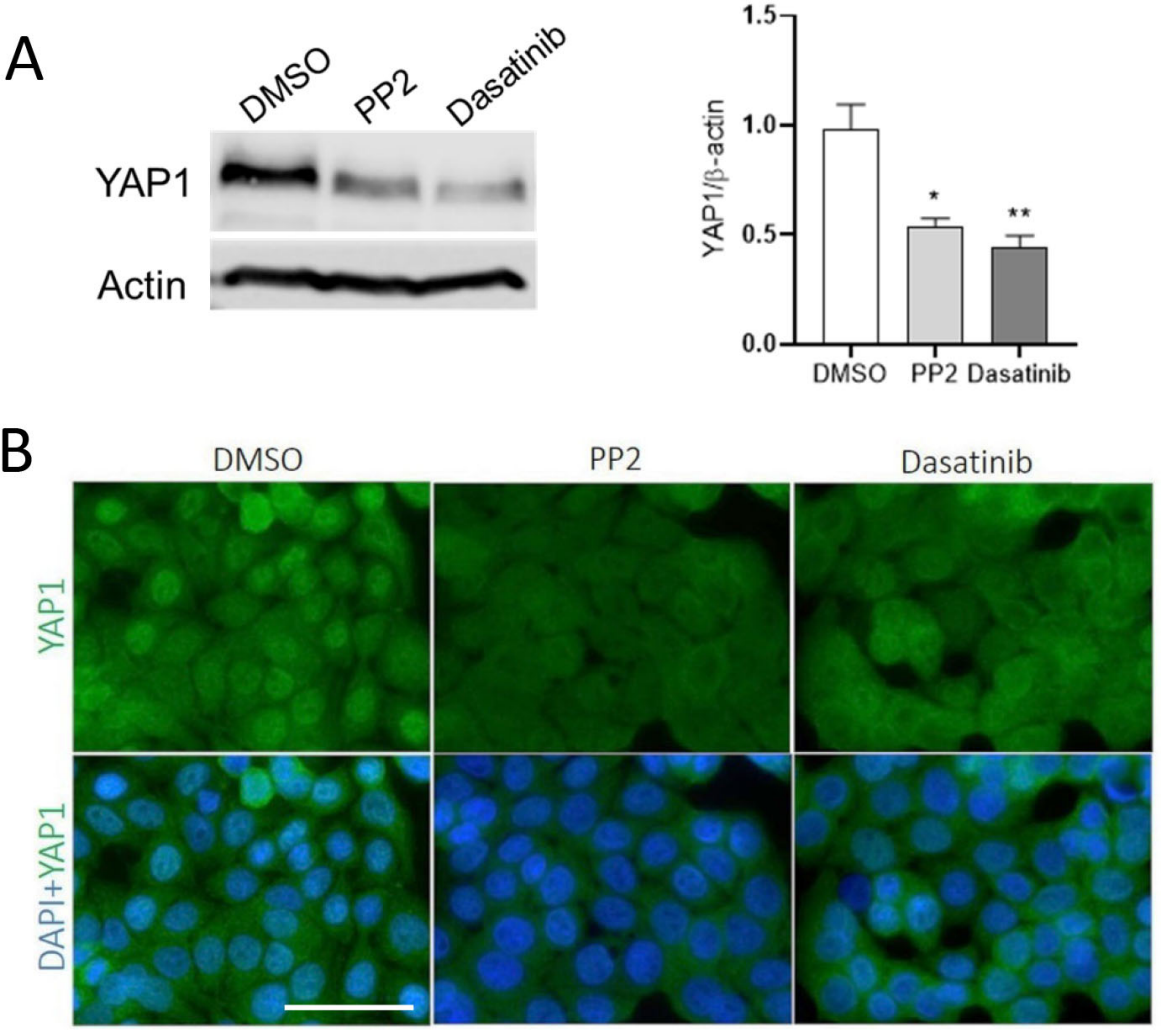
**Reduction of YAP1 in response to SFK inhibition in HT29 cells.** (A) Representative western blot analysis and data compilation showing a reduction of YAP1 expression at the protein level in PP2 and dasatinib treated cells relative to control. β-actin was used as a loading control. Note that since YAP1 was analyzed from the same blot as in Fig. 1B (left set of panels), the β-actin control blot used was the same. Results are expressed as mean±s.e.m.; *n*=3; *P<0.05, **P<0.005. (B) Indirect immunofluorescence staining of HT29 cells showed reduction of nuclear localization of YAP1 protein in a large proportion of PP2 and dasatinib treated cells. DAPI was used for nuclear staining. Scale bar: 50 µm; *n*=3.

**Fig. 3.**
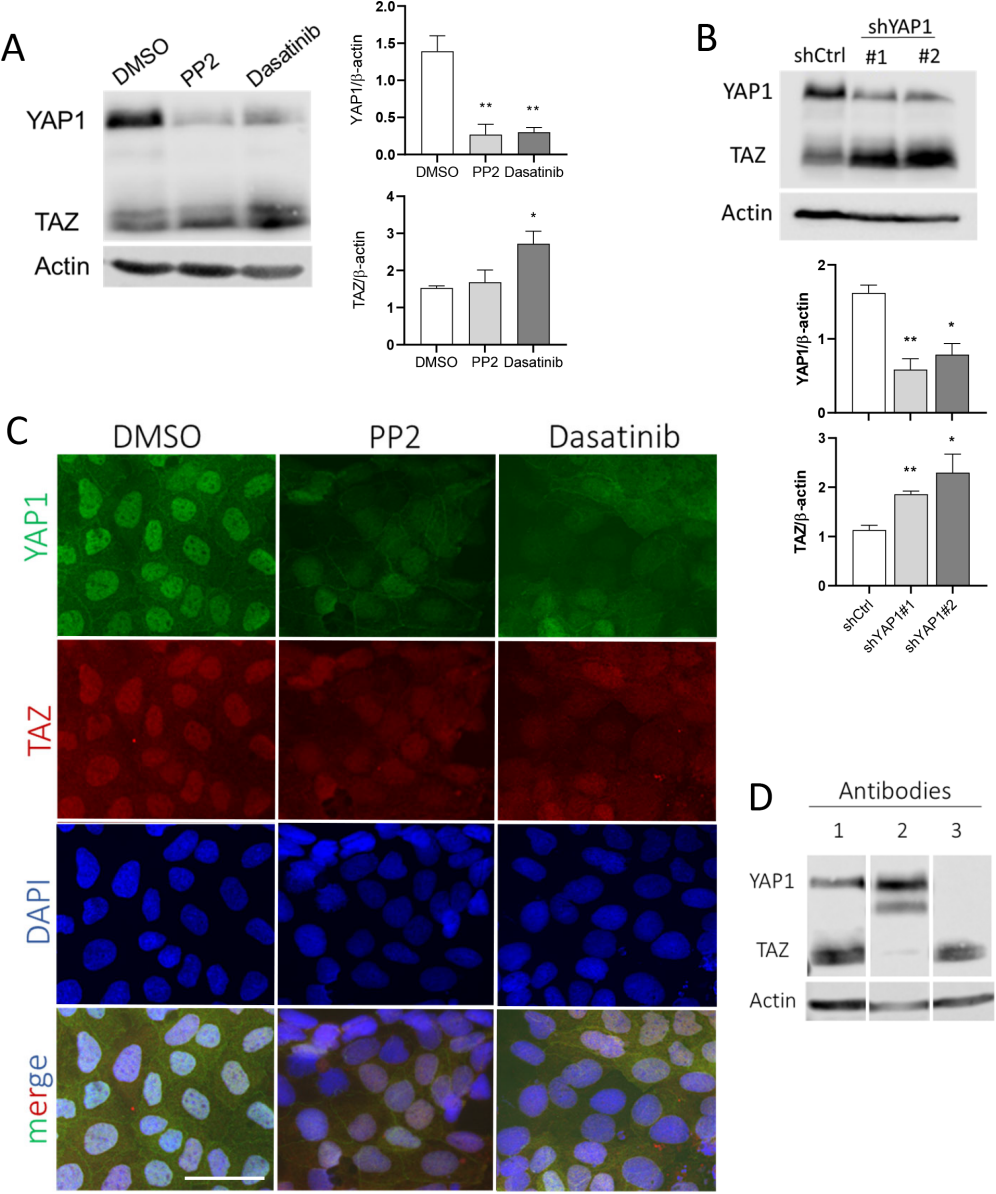
**Reduction of YAP1 in response to SFK inhibition in Caco-2/15 cells.** (A) Representative western blot analysis and data compilation showing a reduction of YAP1 and slight increase of TAZ expression at the protein level in PP2 and dasatinib treated cells relative to control. β-actin was used as a loading control. Note that since YAP1 was analyzed from the same blot as in Fig. 1B (right set of panels), the β-actin control blot used was the same. (B) Representative western blot analysis and data compilation demonstrating the increased expression of TAZ in YAP1 knockdown Caco-2/15 cells compared with control (shLUC). Results are expressed as mean±s.e.m.; *n*=3; *P<0.05, **P<0.005. (C) Indirect immunofluorescence staining of Caco-2/15 cells showing a reduction of nuclear localization of YAP1 protein in a large proportion of PP2 and dasatinib treated cells (upper row panels). Nuclear localization of TAZ protein was reduced in treated cells with SFK inhibitors compared with control (second row panels). DAPI was used for nuclear staining (third row panels) and YAP1, TAZ and DAPI merged (lower row panels). Scale bar: 50 µm; *n*=3. (D) Characterization of the antibodies used for YAP1 (D8H1X, line 2) and TAZ (M2-616, line 3) immunostaining in C as compared to the YAP1/TAZ antibody (D24E4) used for western blot.

Based on previous studies that showed a crosstalk between SFKs and the Hippo pathway and its effectors YAP1 and TAZ in various cell models, we tested the effect of SFK inhibition on YAP1/TAZ expression in HT29 and Caco-2/15 cells. In HT29, only YAP1 was found to be expressed (Fallah and Beaulieu, 2020) and the inhibition of SFKs with PP2 or dasatinib resulted in a reduction of its overall expression after 5 days (Fig. 2A). Cell distribution of YAP1 was also investigated by indirect immunofluorescence. Since HT29 cells tend to strongly aggregate after SFKi treatment, as reported previously (Nam et al., 2002), only short term treated cells were stained for YAP1 detection. As shown in Fig. 2B, even after only an overnight treatment, the nuclear staining of YAP1 was reduced with PP2 and dasatinib as compared with the control. In Caco-2/15, YAP1 and TAZ are both constitutively expressed. SFKi treatments resulted in a reduction of YAP1 in PP2 and dasatinib treated cells compared with control cells, whereas TAZ expression was not reduced by PP2 and even increased in dasatinib treated cells (Fig. 3A). To determine whether TAZ elevation was related to SFK inhibition or YAP1 reduction, YAP1 was knocked down in Caco-2/15 cells using shRNA. The results showed a significant increase of TAZ expression in YAP1 abolished Caco-2/15 cells compared with the control (Fig. 3B). For immunofluorescence, we used YAP1 and TAZ specific antibodies (Fig. 3D). A significant reduction of the nuclear staining was noted for YAP1 with both SFKis while TAZ staining was only slightly decreased relative to the control (Fig. 3C).

**Fig. 4.**
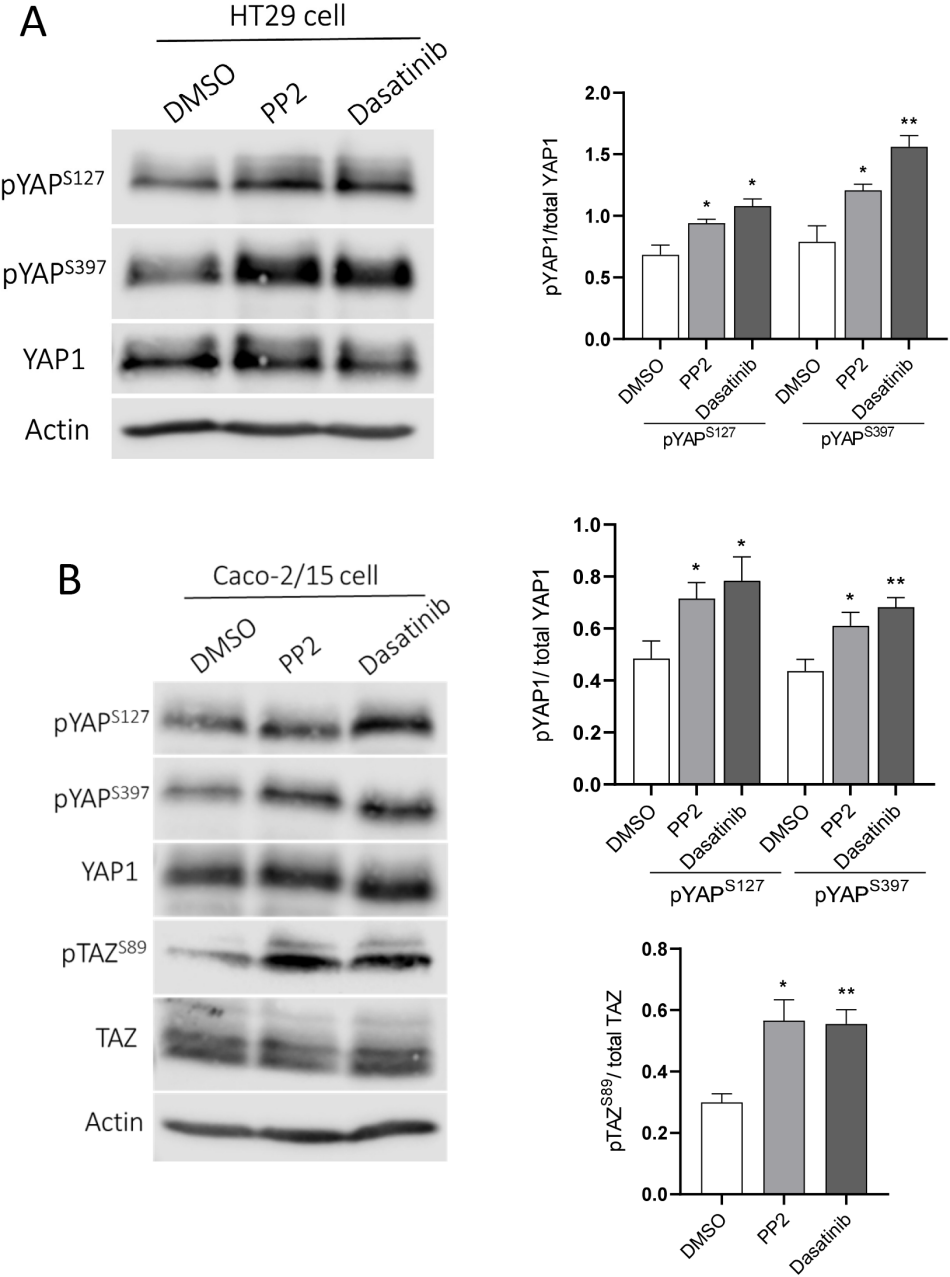
**Inhibition of SFK induces YAP1/TAZ phosphorylation.** (A) Representative western blot analysis and data compilation demonstrating increased levels of pYAP-S127 and pYAP-S397 in HT29 cells treated with PP2 and dasatinib relative to DMSO control. (B) Representative western blot analysis and data compilation demonstrating increased levels of pYAP-S127, pYAP-S397 and pTAZ-S89 in PP2 and dasatinib treated Caco-2/15 cells relative to DMSO. β-actin was used as a loading control. Results are expressed as mean±s.e.m.; *n*=3; *P<0.05, **P<0.005.

One of the mechanisms proposed for YAP1/TAZ regulation by SFKs is their control on the Hippo pathway effector LATS1/2 kinase where SFK inhibition can result in increased YAP1/TAZ phosphorylation in serine residues that leads to their cytoplasmic sequestration and/or proteasomal degradation (Lamar et al., 2019). Considering that we observed a reduction of YAP1 expression in HT29 and Caco-2/15 cell extracts from SFKi treated cells as well as a reduction in nuclear staining, we chose to focus on the phosphorylation of YAP1 (pYAP1) on serine 127 and serine 397 involved in YAP1 cytoplasmic sequestration and degradation, respectively. Relative levels of pYAP1-S127 and pYAP1-S397 were evaluated in HT29 and Caco-2/15 cells after a 4 h treatment with PP2, dasatinib or DMSO by WB analysis. Increases in pYAP1-S127 and pYAP1-S397 were observed in SFK inhibited cells for both HT29 (Fig. 4A) and Caco-2/15 cells (Fig. 4B) relative to the control. Furthermore, phosphorylation of TAZ associated with cytoplasmic sequestration (pTAZ-S89) was also increased in Caco-2/15 treated cells with SFKis compared with the control (Fig. 4B).

### SFKs control the differentiation of intestinal epithelial cells through the release of YAP1 dependent repression of the pro-differentiation transcription factors CDX2 and HNF4α

**Fig. 5.**
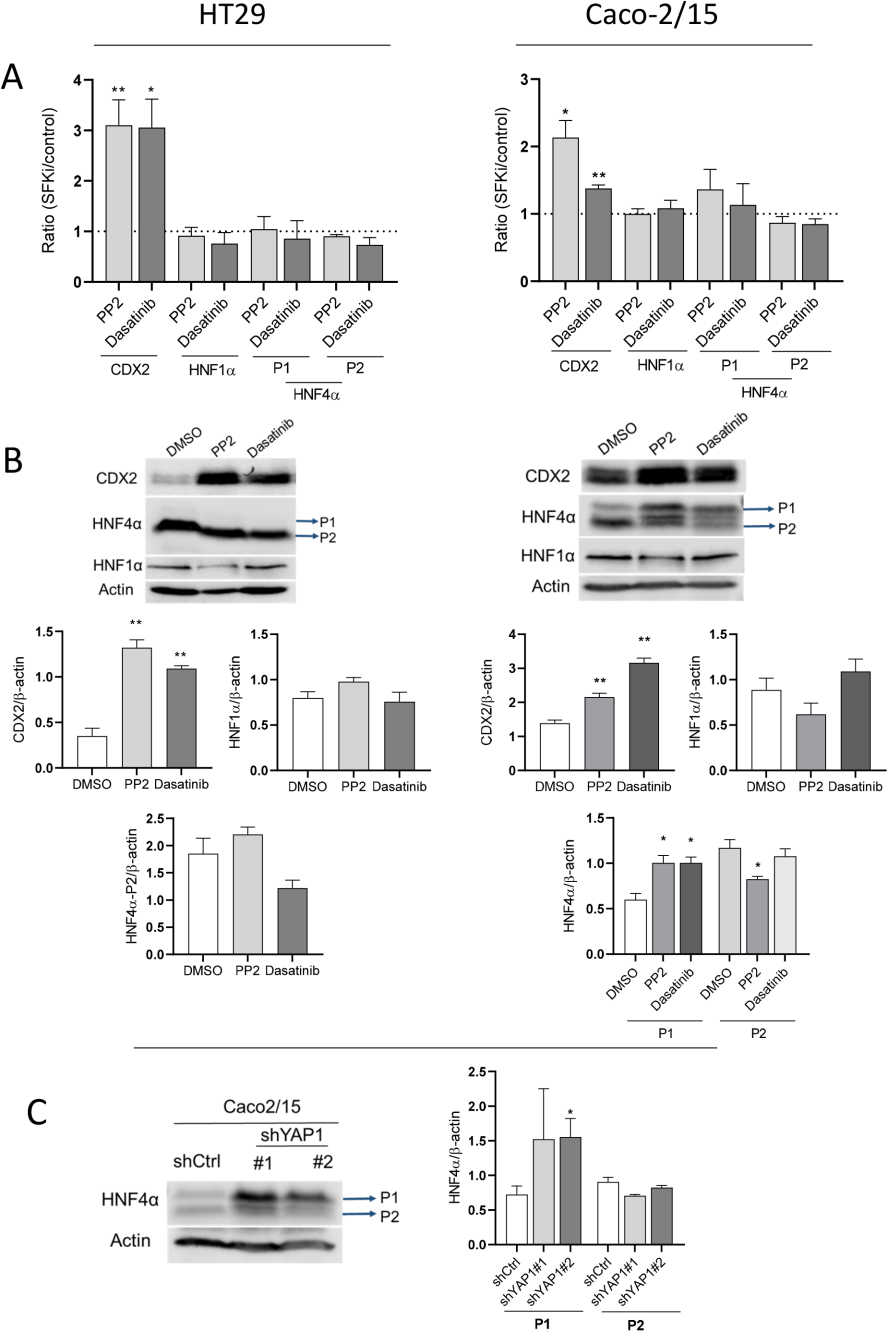
**Modulation of the expression of the transcription factors CDX2, HNF4α and HNF1α in response to SFK inhibition in HT29 and Caco-2/15 cells.** (A) qPCR analysis showing the mRNA levels of expression for the three transcription factors in PP2 and dasatinib treated HT29 and Caco-2/15 cells. (B) Representative western blot analyses and data compilations showing higher expression of CDX2 protein in PP2 and dasatinib treated HT29 cells relative to the control while both CDX2 and P1 isoforms of the HNF4α protein were found to be increased in PP2 and dasatinib treated Caco-2/15 cells relative to DMSO. (C) Representative western blot analysis and data compilation showing higher expression of P1 isoforms of the HNF4α protein in YAP1 ablated Caco-2/15 cells compared with control. β-actin was used as a loading control. Results are expressed as mean±s.e.m.; *n*=3; *P<0.05, **P<0.005.

The central role of some specific transcription factors such as CDX2, HNF1α and HNF4α on the regulation of intestinal cell-specific gene expression associated with terminal differentiation is well documented. While below the detection level in normal HT29, CDX2 expression was induced in YAP1 knockdown HT29 cells and was responsible for absorptive and goblet cell differentiation (Fallah and Beaulieu, 2020). Expression of CDX2, HNF1α and HNF4α was first analyzed at the transcript level in HT29 and Caco-2/15 cells treated with SFKis relative to controls. CDX2 was found to be induced in both cell lines with either PP2 or dasatinib (Fig. 5A). Transcript levels of HNF1α were not found to be modulated by SFKis nor were HNF4α P1 (including α1 to α6 isoforms) and P2 (including α7 to α12 isoforms) classes (Babeu et al., 2018). The induction of CDX2 in SFKis treated cells was also confirmed at the protein level for HT29 and Caco-2/15 (Fig. 5B) while the levels of HNF1α remained unaltered under the same conditions. For HNF4α, only the P2 class isoforms were detected in HT29 and their expression was not affected in the SFKis treated cells (Fig. 5B). In Caco-2/15, a reduction of the P2 class of isoforms was observed after PP2 treatment while the expression of P1 isoforms were increased in PP2 and dasatinib-treated cells as compared with DMSO controls (Fig. 5B). To understand whether the increase in P1 isoforms resulted from YAP1 reduction, the expression of the P1 proteins was measured in YAP1 knockdown Caco-2/15 cells. Expression of P1 isoforms of HNF4α protein was increased significantly in shYAP1 expressing Caco-2/15 cells compared with the control (shLUC); however, P2 isoforms remained unchanged (Fig. 5C).

### TAZ knockdown impairs differentiation of intestinal absorptive cells

**Fig. 6.**
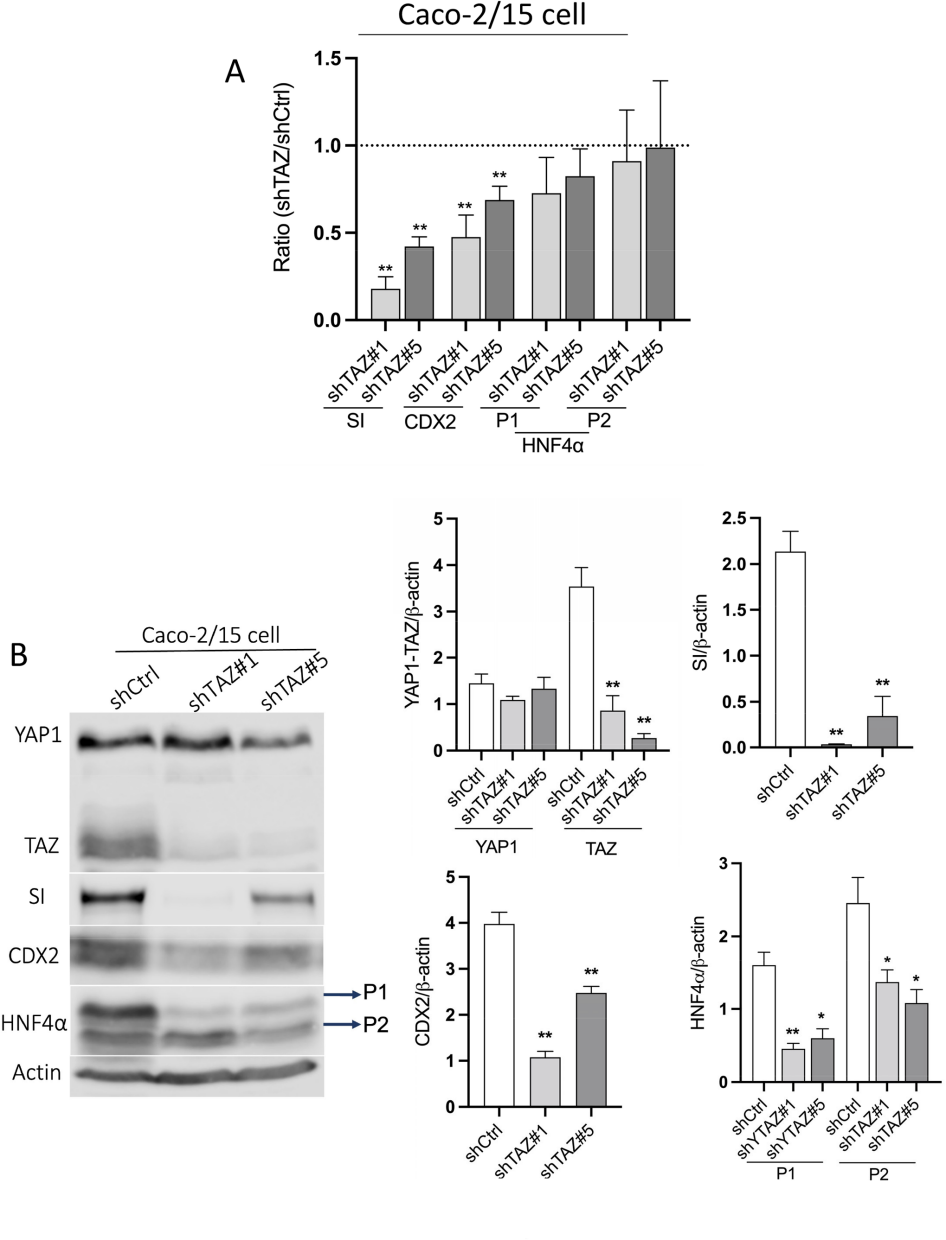
**The effect of TAZ knockdown on absorptive cell differentiation in Caco-2/15 cells.** (A) qPCR analysis showing the transcriptional level of SI, CDX2 and the P1 and P2 classes of HNF4α in TAZ knockdown Caco-2/15 cells compared with control. (B) Representative western blot analyses and data compilations showing a reduction of TAZ, SI and CDX2 as well as the two classes of HNF4α in TAZ knockdown Caco-2/15 cells relative to the control. Results are expressed as mean±s.e.m.; *n*=3; *P<0.05, **P<0.005.

Given that SFK inhibition and YAP1 knockdown in Caco-2/15 cells were accompanied by an increase in the expression of TAZ protein, the net influence of TAZ on the differentiation of intestinal epithelial cells was investigated in Caco-2/15 cells. The expression of TAZ was knocked down in Caco-2/15 cells using shRNA. As expected, a sharp reduction in TAZ protein was observed while the expression of the YAP1 protein remained stable (Fig. 6). To study the effect of TAZ knockdown in absorptive cell differentiation, the expression of SI was detected at both transcript and protein levels. Contrary to YAP1, TAZ knockdown led to a reduction of SI expression in Caco-2/15 cells relative to the shGFP control (Fig. 6A,B). Since in the present study, CDX2 and P1 isoforms of HNF4α have been identified as important factors participating in YAP1 related epithelial cell differentiation, their expression was measured in TAZ knockdown Caco-2/15 cells. The results showed that CDX2 was reduced in TAZ knockdown cells, while HNF4α was not altered at the transcript level (Fig. 6A). Furthermore, the expressions of CDX2 as well as both classes of HNF4α isoforms were decreased at the protein level in TAZ knockdown Caco-2/15 cells compared with the control (Fig. 6B).

## DISCUSSION

The differentiation of intestinal epithelial cell lineages is a tightly regulated process involving the contribution of various signaling pathways such as Wnt/β-catenin, Notch and Hippo and their downstream effectors (Tian et al., 2015; Spit et al., 2018; Beumer and Clevers, 2021) as well as epigenetic mechanisms (Roostaee et al., 2016). In the present study, we have investigated the involvement of SFKs on the expression of YAP1/TAZ in the context of intestinal goblet and absorptive cell differentiation. Indeed, it has been shown previously that SFKs, namely Src, can stimulate YAP1/TAZ expression in cancer cells of various types (Lamar et al., 2019). In another study, remodeling of the extracellular matrix in a model of colonic regeneration was linked to an increase in Src signaling and YAP1/TAZ activation (Yui et al., 2018). These studies are consistent with the involvement of YAP1/TAZ in intestinal regeneration (Barry et al., 2013; Gregorieff et al., 2015; Byun et al., 2017) and the fact that the activation of SFKs as well as YAP1/TAZ is regulated by several factors such as mechanical stress, including cell–cell contact, cell–matrix adhesion, extracellular stiffness, etc. (Zhao et al., 2007; Boopathy and Hong, 2019). However, previous studies from our group have found that SFK inhibition in intestinal epithelial cells accelerated the expression of terminal differentiation markers in Caco-2/15 cells (Seltana et al., 2013) while knockdown of YAP1 stimulated both goblet and absorptive cell differentiation in HT29 (Fallah and Beaulieu, 2020) suggesting that the SFK-YAP1/TAZ axis may also be involved in the repression of intestinal cell maturation. In this study, we report that SFKs exert an inhibitory effect on goblet and absorptive cell differentiation by activating YAP1, which acts through the repression of the pro-differentiation transcription factors CDX2 and the HNF4α P1 family. Furthermore, our results indicate that TAZ is not affected by SFKi and has no apparent influence on intestinal differentiation, suggesting that the two paralogs are not regulated nor acting through the same mechanisms.

It is noteworthy that YAP1 was initially cloned as an interacting protein of c-Yes which is a member of the SFKs but was shown later that its phosphorylation on tyrosine was by Src while Yes had a minimal effect on YAP1 phosphorylation and nuclear localization (Zaidi et al., 2004; Kim and Gumbiner, 2015). Herein we have tested the two pharmacological inhibitors of SFK, PP2 and dasatinib, in HT29 cells, which resulted in a higher expression of both absorptive (SI and DPPIV) and goblet (MUC2 and TFF3) cell markers. This result was confirmed with Caco-2/15 cells with an increase in expression of SI and DPPIV indicating that SFKs negatively regulate both goblet and absorptive cell differentiation. Incidentally, PP2 treatment of Caco-2/15 cells at confluency was reported to be accompanied by acceleration of absorptive cell differentiation and elevation of SI expression (Seltana et al., 2013). Nam and colleagues also have reported that an increased level of E-cadherin, which is a marker of epithelial cell differentiation, and α/β/γ catenin proteins was observed by treatment of HT29 cells with the PP2 inhibitor. In addition, cell adhesiveness was increased by inhibiting SRC activity (Nam et al., 2002). E-cadherin is a component of the adherens junction in epithelial tissue. It is expressed strongly at the upper part of the crypt, but its expression is reduced toward the base of the crypt (Escaffit et al., 2005). Treatment of pancreatic adenocarcinoma cells with dasatinib also resulted in increased total and membranous E-cadherin/β-catenin levels (Dosch et al., 2019). It has been suggested that Src kinase downregulates the expression of E-cadherin through the pro-EMT transcription factor Slug, which is a negative transcriptional regulator of E-cadherin (Dosch et al., 2019). Treatment of Caco-2/15 and HT29 cells with PP2 and dasatinib in the present work was also accompanied by partial abolition of the YAP1 protein, which suggests a regulatory effect of SFKs on YAP1 activity. Recent studies have demonstrated a high activity of SFKs and YAP1/TAZ in cancers. Lamar and colleagues have reported that increased SRC activity may be the driver of high activity of YAP1/TAZ in human cancers (Lamar et al., 2019). This group has shown that inhibition of Src activity using dasatinib was accompanied by a reduction in YAP1/TAZ activity, tumor growth and metastasis. On the contrary, the expression of constitutive active Src under the form of the Src Y527F mutant, led to elevated levels of YAP1/TAZ and of its target genes in several human and mouse breast cancer and melanoma cell lines (Lamar et al., 2019). Unlike YAP1, increased levels of TAZ protein in Caco2/15 cells treated with SFKis suggests that TAZ expression is regulated by a different mechanism. This possibility has been confirmed by TAZ elevation after YAP1 abolition using shRNA in agreement with previous studies showing that in the mouse, YAP1 negatively regulates the abundance of TAZ protein post-transcriptionally by proteasomal degradation, whereas TAZ expression has no effect on YAP1 abundance (Finch-Edmondson et al., 2015). Therefore, an increase in TAZ expression in Caco-2/15 cells can be related to the partial disappearance of the YAP1 protein. The reason for a stronger increase in TAZ abundance in shYAP1 cells as compared with SFKi-treated cells is not clear but it could be speculated that the constitutive abolition of YAP1 in shYAP1 cells may be more favorable for the reduction of TAZ degradation than the transient YAP1 reduction resulting from SFKi treatment. Our results for indirect IF staining of YAP1 in HT29 and Caco-2/15 cells showed an observable disappearance of nuclear localization of YAP1 protein, which is accompanied by an increase of cytoplasmic staining in PP2 and dasatinib treated cells. Treatment of MCF-10A cells with PP2 was accompanied by an abolition of nuclear localization of YAP1 and an elevation of phosphorylated YAP1 (S127) but TAZ localization was not investigated (Kim and Gumbiner, 2015). A reduction in nuclear localization of TAZ was observed by indirect immunofluorescence in Caco-2/15 cells, but an increase in cytoplasmic staining was not observed although an elevation of TAZ expression in SFKi treated cells was consistent when analyzed by western blot. This inconsistency could be related to the difficulty of detecting TAZ by immunofluorescence (Kingston et al., 2019).

There are various mechanisms by which SFKs and Src kinase could regulate YAP1/TAZ activity. SFKs and Src kinase can directly affect YAP1/TAZ activity by increasing their stability and transcriptional activity through phosphorylation at tyrosine Y357 and Y316, which promotes their nuclear localization and stabilization, respectively (Taniguchi et al., 2015; Li et al., 2016; Byun et al., 2017; Sugihara et al., 2018). On the other hand, Src kinase phosphorylates LATS1/2 in several tyrosine residues which suppresses its activity and therefore induces YAP1/TAZ activity (Si et al., 2017). Indeed, LATS1/2 kinases are responsible for YAP1 and TAZ phosphorylation in serine 127 and serine 89, respectively, which leads to their cytoplasmic restraining and inactivation as well as on serine 397 of YAP1, which leads to its recognition by SCF (β-TrCP), ubiquitination and proteasomal degradation (Zhao et al., 2007; Lei et al., 2008). In addition, it has been demonstrated that the inhibitory effect of PP2 on YAP1 is abolished by knockdown of the LATS1/2 kinases (Kim and Gumbiner, 2015). LATS1/2 kinases can be phosphorylated on serine 909 and/or threonine 1079 by MST1/2 kinases or other regulators like MAP4K, which leads also to their activation (Ni et al., 2015; Si et al., 2017). Considering that inhibition of SFKs induced a sharp reduction of YAP1 protein in intestinal cells, we thus explored the LATS1/2 kinase-related pathway. Our results showed that pYAP-S127, pYAP-S397 and pTAZ-S89 were increased after 4 h of PP2 and dasatinib treatment, a result consistent with the partial abolition of the YAP1 protein in both HT29 and Caco-2/15 cells in long-term (5 days) PP2 and dasatinib treated cells. The lack of net effect on TAZ protein levels, albeit an increase in S89 phosphorylation, can be attributed to the increase in its expression resulting from YAP1 abolition as mentioned above.

Next, the mechanism by which the SFK-YAP1 axis appears to restrain intestinal differentiation was further investigated. In HT29, we previously showed that CDX2 was expressed in YAP1 knockdown cells while it remained below detection levels in wild-type cells and confirmed that the goblet and absorptive cell differentiation triggered by YAP1 ablation was abolished by CDX2 knockdown (Fallah and Beaulieu, 2020). CDX2 plays a significant role in the differentiation of small and large intestinal epithelial cells. Its expression rises from the crypt to the lumen (Silberg et al., 2000; Benoit et al., 2010) and its ectopic expression in crypt-like cells was shown to initiate an enterocytic differentiation program (Suh and Traber, 1996; Escaffit et al., 2006; Benoit et al., 2010). It is also noteworthy that interaction of CDX2 with the promoters of SI, MUC2 and TFF3 has been reported previously (Boudreau et al., 2002; Mesquita et al., 2003; Yamamoto et al., 2003; Shimada et al., 2007; Boyd et al., 2010). Significant increases in CDX2 were noted at both protein and transcript levels in Caco-2/15 cells after treatment with SFKis, as expected (Seltana et al., 2013). The level of HNF1α, another key transcription factor involved in SI expression (Boudreau et al., 2002) also found to be modulated in Caco-2/15 cells after PP2 treatment (Seltana et al., 2013), remained unchanged under our conditions (earlier stage and new anti-HNF1α antibody, the original one being discontinued). HNF4α is another transcription factor that plays a key role in enterocyte differentiation in conjunction with CDX2 (San Roman et al., 2015). It has been shown in the intestinal epithelium that HNF4α has two classes of isoforms, P1 and P2, where only P1 isoforms are associated with cell differentiation (Babeu et al., 2018). In HT29, only P2 isoforms were detected while in Caco-2/15 cells, the protein amounts of P1 isoforms were increased after SFKi treatment. To verify whether the increase in levels of P1 class HNF4α isoforms was related to YAP1 abolition, HNF4α was tested in YAP1 knockdown Caco-2/15 cells which confirmed that P1 isoforms of HNF4α protein are regulated by YAP1. The regulation appears to occur at the protein level since transcripts were not modulated. Incidentally, it has been reported that YAP1 can negatively regulate HNF4α expression through ubiquitination and proteasomal degradation in hepatocellular carcinoma (HCC) cells (Cai et al., 2017). It is interesting to note that CDX2 and HNF4α regulate YAP1 expression by interacting with its promoter (Larsen et al., 2019).

Finally, one apparent puzzling set of findings from this work is the fact that YAP1 and TAZ do not seem to be regulated by the same mechanism in response to SFK inhibition and, even more importantly, do not play the same role on the regulation of intestinal cell differentiation, YAP1 acting as a potent inhibitor through the repression of CDX2 and HNF4α expression while TAZ knockdown appears to alter the expression of these pro-differentiation transcription factors, at least in Caco-2/15 cells. However, a potential specific requirement of TAZ for intestinal differentiation is unlikely considering two independent observations: First, in contrast to its upregulated expression in YAP1 knockdown Caco-2/15 cells, SFKi treatment in these cells stimulate absorptive cell differentiation without significantly stimulating TAZ expression. Second, HT29 cells, which lack TAZ expression, can be triggered toward absorptive and goblet cell differentiation after YAP-1 knockdown. In this context, our finding may trigger further investigation into the functional differences between the two paralogs in the intestinal epithelium. Indeed, on one hand, YAP1 and TAZ are considered to act similarly (Meng et al., 2016; Boopathy and Hong, 2019; Pocaterra et al., 2020). For instance, the possibility that Taz may compensate for Yap in the intestinal crypts of Yap knockout mice has been raised (Barry et al., 2013). On the other hand, despite their similarities, there is growing evidence that they can be distinguished by structural and functional aspects (Jeong et al., 2021; Reggiani et al., 2021).

In summary, the present study revealed that SFKs negatively regulate the differentiation of intestinal absorptive and goblet cells through the upregulation of the Hippo pathway coactivator YAP1, which appears to mainly act through the repression of the key pro-differentiation transcription factor CDX2 and degradation of the P1 class of HNF4α isoforms. On the other hand, its paralog TAZ, for which net expression is upregulated in YAP1 knockdown Caco-2/15 cells, appears to have a different function, which may be related to the recruitment of specific transcription factors as suggested by the inhibition of absorptive cell differentiation in TAZ knockdown cells. Overall, YAP1 and TAZ appear to exert distinct effects on the regulation of intestinal differentiation.

## MATERIALS AND METHODS

### Cell culture and lentivirus-mediated RNA interference

The human colorectal cancer cell lines HT29 and Caco2/15 (Beaulieu and Quaroni, 1991) were provided by A. Quaroni (Cornell University, Ithaca, NY, USA). Cells were cultured in DMEM medium (Life Technologies, Burlington, ON, Canada), supplemented with 10% fetal bovine serum (Wisent, Saint-Jean-Baptiste, QC, Canada), 2 mM GlutaMAX (Life Technologies) and 10 mM HEPES (Wisent) under standard conditions. For confirmation of HT29 and Caco2/15 cell line identities, short-tandem repeat profiling cell authentication was performed. Both cell lines were routinely monitored for mycoplasma contamination. The colorectal cancer cell lines HT29 and Caco-2/15 have been used because of their potential for differentiation (Augeron and Laboisse, 1984; Zweibaum et al., 1985). They were isolated from colorectal cancers but while maintained under certain conditions, they express fetal colonocyte characteristics (Augeron and Laboisse, 1984; Zweibaum et al., 1985; Beaulieu and Quaroni, 1991; Vachon and Beaulieu, 1992; Kitamura et al., 1996; Pageot et al., 2000; Tremblay et al., 2006). Indeed, during mid-gestation, the structure of the colon is similar to that of the small intestine containing the villus, brush border and digestive enzymes (Ménard et al., 2006).

In order to knockdown YAP1, lentivirus sequences containing shRNA targeting YAP1 from Addgene (Watertown, Massachusetts, USA) was obtained as a gift from William Hahn (Rosenbluh et al., 2012) (Addgene plasmids # 42540 and 42541). The sequences were shYAP1#1; 5′-GCCACCAAGCTAGATAAAGAA-3′ and shYAP1#2; 5′-CCCAGTTAAATGTTCACCAAT-3′. In addition, shLUC (Benoit et al., 2012) and shGFP (Groulx et al., 2014) were used as controls. A shRNA targeting WWTR1 (TAZ) was obtained from Sigma-Aldrich (cat #TRCN0000019469 and TRCN0000370007; Oakville, ON, Canada). The sequences were shTAZ#1; 5′-GCGATGAATCAGCCTCTGAATC-3′ and shTAZ#5; 5′-GCGTTCTTGTGACAAGATTATA-3′. Preparation of viruses was performed using HEK293T cells. Caco-2/15 cells were plated in 100 mm dishes one day before transfection. The day after, 50% confluent cells were infected. After 48 h, the infected cells were selected using 10 μg/ml puromycin for 9 days.

### Cell signaling inhibitor treatments

HT29 and Caco-2/15 cells were incubated with SFK inhibitors including PP2 (20 µM, Abcam, Toronto, ON, Canada) and dasatinib (10 µM, Abcam) at day −2 of confluence (80% confluent) for 40 min, overnight or 5 days, depending on the experiment. Stock solutions of PP2 and dasatinib were prepared in DMSO (Sigma-Aldrich, Oakville, ON, Canada). Controls consisted of DMSO only. The inhibitors and DMSO were added to the medium and renewed daily. Finally, cells were processed for immunofluorescence or harvested for total RNA and protein extraction.

### Antibodies

In the present study, mouse monoclonal anti-MUC2 (ab11197, 1/500 WB and 1/100 IF, Abcam), rabbit monoclonal anti-TFF3 (ab108599, 1/1500 WB, Abcam), mouse monoclonal anti-SI ([53]; HSI-4/34 or Caco-3/73, 1/100 WB), rabbit monoclonal anti-DPPIV or CD26 [EPR20819] (ab215711, 1/2000 WB, Abcam), rabbit monoclonal anti-YAP/TAZ (D24E4, 1/1500 WB, Cell Signaling Technology, Danvers, MA, USA), rabbit monoclonal anti-YAP (D8H1X, 1/1000 WB, 1/150 IF, Cell Signaling Technology), mouse monoclonal anti-TAZ (M2-616, 1/300 IF, BD Biosciences, NJ, USA) mouse monoclonal anti-CDX2-CD88 (MU392A-UC, 1/700, BioGenex, Freemont, CA, USA), mouse monoclonal anti-HNF1α [F-7] (sc-393925, 1/300 WB, Santa Cruz Biotechnology, Dallas, TX, USA), goat polyclonal anti-HNF4α [C-19] (sc-6556, 1/600 WB, Santa Cruz Biotechnology) and anti-β-actin (MAB1501, 1/20,000, Millipore, Etobicoke, ON, Canada) were used as primary antibodies. In addition, AlexaFluor 488 or 594 goat anti-mouse (A11017, A11072, 1/400; Thermo Fisher Scientific, Ottawa, ON, Canada) and goat anti-rabbit (A11070, A11072, 1/400), ECL HRP-linked anti-mouse (NA931V, 1/4000, GE Healthcare, Mississauga, ON, Canada) and anti-rabbit (NA934V, 1/4000) and HRP-linked bovine anti-goat (sc-2350, 1/4000, Santa Cruz Biotechnology) were used as secondary antibodies.

### Western blot analysis

Laemmli 1× buffer was used for protein extraction of harvested cells. Then the lysed cells were sonicated and centrifuged at 13,000 RPM for 15 min at 4°C. Finally the supernatants were harvested and protein concentrations were determined using the Lowry method with Folin phenol reagent and with BSA as the protein standard (Lowry et al., 1951). Then the western blot analysis was performed as described previously (Fallah and Beaulieu, 2020). Briefly, equal amounts (50 µg) of each reduced (5% β-mercaptoethanol) protein sample were migrated through sodium dodecyl sulfate-polyacrylamide gel electrophoresis (SDS-PAGE) and 2% vertical agarose gel electrophoresis. Samples were transferred from gels onto nitrocellulose membranes (GE Healthcare, Mississauga, ON, Canada) and nonspecific binding sites were blocked using 5% fat-free milk. The membranes were incubated overnight with primary antibodies at 4°C and then 1 h with horseradish peroxidase-conjugated secondary antibodies at room temperature. Finally, HRP positive bands were detected using the enhanced chemiluminescence (ECL) method (Millipore, Etobicoke, ON, Canada). LI-COR Biosciences/Odyssey Imager and Image Studio Lite 5.2 software were used for observing the bands and taking images. ImageJ (Rasband, 1997-2018) (National Institute of Health, Bethesda, MD, USA) was used for scanning and band quantitation.

### RNA extraction, reverse transcriptase and quantitative RT-PCR

Cells were lysed for RNA extraction using RiboZol (VWR Life Science, Solon, OH, USA). The extraction of RNA was performed according to the manufacturer's instructions (VWR Life Science). Reverse transcription of RNA was performed using the Omniscript RT kit (Qiagen, Germantown, MD, USA). Green-2-Go qPCR low ROX Master Mix (BioBasic, Markham, ON, Canada) was used for quantitative polymerase chain reaction (qPCR). RNA extraction, reverse transcription and qPCR assays were performed as described previously (Dydensborg et al., 2006). The primers used for qPCR included: CDX2: forward 5′-GAGTGGTGTACACGGACCAC-3′ and reverse 5′-TTTCCTCTCCTTTGCTCTGC-3′; HNF1α: forward 5′-CCGCAGACTATGCTCATCAC-3′ and reverse 5′-GCTGAGTCTGAGCTCTGGT-3′; HNF4α: P1 forward 5′-GGAATTTGAGAATGTGCAGGTGTTG-3′ and reverse 5′-TGAGGTTGGTGCCTTCTGATG-3′; HNF4α P2: forward 5′-GCCATGGTCAGCGTGAAC-3′ and reverse 5′-CGTTGAGGTTGGTGCCTTCT-3′; MUC2: forward 5′-CATCACATTCATGCCCAATG-3′ and reverse 5′-CAGCTCTCGATGTGGGTGTA-3′; SI: forward 5′-GAGGACACTGGCTTGGAGAC-30 and reverse 5′-ATCCAGCGGGTACAGAGATG-3′; DPPIV: forward 5′-AAGTGGCGTGTTCAAGTGTG-3′ and reverse 5′-CAGGGCTTTGGAGATCTGAG-3′.

Gene expression was normalized using RPLPO (Dydensborg et al., 2006) and relative quantification was calculated according to the Pfaffl equation (Pfaffl et al., 2004).

### Indirect immunofluorescence staining

HT29 and Caco2/15 cells were seeded on coverslips and cell culture Lab-Tek chamber slides (Nalgen Nunc, Naperville, IL, USA), respectively. After treatment with PP2, dasatinib and DMSO as a control, cells were fixed with 4% PFA for 15 min at +4°C. After rinsing with PBS-glycine and permeabilization with 0.1% triton, cells were incubated with 10% goat serum as blocker for 1 h at room temperature and then with the primary antibody overnight at +4°C. Secondary antibody, DAPI and Evans blue were utilized for immunofluorescence, nuclear and background staining, respectively. A Leica DM RXA microscope was used for observing the cells and taking images. The images were acquired using MetaMorph software (Universal Imaging Corporation, West Chester, PA, USA).

### Statistical analysis

For data preparation and statistical analysis including the two-tailed Student's *t*-test, Graph Pad Prism 8.3 (Graph Pad Software; San Diego, CA, USA) was used. A *P*-value below <0.05 was considered significant in all analyses unless otherwise specified. All experiments were independently repeated at least three times.
